# Experimental optimization of probe length to increase the sequence specificity of high-density oligonucleotide microarrays

**DOI:** 10.1186/1471-2164-8-373

**Published:** 2007-10-16

**Authors:** Shingo Suzuki, Naoaki Ono, Chikara Furusawa, Akiko Kashiwagi, Tetsuya Yomo

**Affiliations:** 1 Department of Bioinformatics Engineering, Graduate School of Information Science and Technology, Osaka University, 2-1 Yamadaoka, Suita, Osaka 565-0871, Japan; 2 Complex Systems Biology Project, ERATO, Japan Science and Technology Corporation, Osaka University, 2-1 Yamadaoka, Suita, Osaka 565-0871, Japan; 3 Graduate School of Frontier Biosciences, Osaka University, 1-3 Yamadaoka, Suita, Osaka 565-0871, Japan

## Abstract

**Background:**

High-density oligonucleotide arrays are widely used for analysis of genome-wide expression and genetic variation. Affymetrix GeneChips – common high-density oligonucleotide arrays – contain perfect match (PM) and mismatch (MM) probes generated by changing a single nucleotide of the PMs, to estimate cross-hybridization. However, a fraction of MM probes exhibit larger signal intensities than PMs, when the difference in the amount of target specific hybridization between PM and MM probes is smaller than the variance in the amount of cross-hybridization. Thus, pairs of PM and MM probes with greater specificity for single nucleotide mismatches are desirable for accurate analysis.

**Results:**

To investigate the specificity for single nucleotide mismatches, we designed a custom array with probes of different length (14- to 25-mer) tethered to the surface of the array and all possible single nucleotide mismatches, and hybridized artificially synthesized 25-mer oligodeoxyribonucleotides as targets in bulk solution to avoid the effects of cross-hybridization. The results indicated the finite availability of target molecules as the probe length increases. Due to this effect, the sequence specificity of the longer probes decreases, and this was also confirmed even under the usual background conditions for transcriptome analysis.

**Conclusion:**

Our study suggests that the optimal probe length for specificity is 19–21-mer. This conclusion will assist in improvement of microarray design for both transcriptome analysis and mutation screening.

## Background

High-density oligonucleotide microarrays allow analysis of the genome-wide expression of genes in living organisms [1] and for genome-wide screens of genetic variation and disease-causing mutations [2,3]. The Affymetrix GeneChip system is one of the most commonly used high-density oligonucleotide microarray systems because each probe is synthesized in the precise location and millions of probes can be contained on an array. In the Affymetrix GeneChip system, the expression of each transcript is measured using a set of probe pairs, *i.e*., a perfect match (PM) probe that matches a fragment of the corresponding gene exactly and a mismatch (MM) probe containing a single nucleotide mismatch in the center. It is generally assumed that the MM probe provides a measure of cross-hybridization to corresponding PM probes, and thus subtracting the signal intensities of MM probes from those of PM probes allows canceling of the effect of cross-hybridization [4].

However, it has been pointed out that around 30% of probe pairs consistently give negative signals, which means that the difference between PM and MM probe intensity does not always reflect the true target amounts [5,6]. This contradiction of PM and MM probe intensities is the main factor making expression analysis unreliable, especially when the target concentration is low. Such contradictions will occur when, for example, the difference in the amount of target specific hybridization between PM and MM probes is smaller than the variance in the amount of cross-hybridization. Therefore, to improve the measurement of target amounts using the pairs of PM and MM probes, one possible strategy is to enhance the specificity for single nucleotide mismatches, *i.e*., changes in signal intensity caused by a single nucleotide mismatch. In the present study, we focused on this discrimination capability of single nucleotide mismatches and performed evaluation using the signal intensity ratio of PM to MM probes. The enhancement of specificity for single nucleotide mismatches is not only required for the improvement of the original Affymetrix analysis method (MAS5.0), it is also useful for development of other analysis models, such as dChip [7] or Robust Microarray Analysis (RMA) [8,9], which do not make use of MM probes, as it will reduce noise from targets of similar sequence to the desired target sequence. The specificity is also important for analysis of single nucleotide polymorphisms (SNPs) using microarray technology [10,11].

Several previous studies on microarray technology investigated the specificity for single nucleotide mismatches experimentally. For example, with regard to probe length, a previous study was performed using an oligonucleotide microarray where 25-, 30-, and 35-mers were printed on glass slides [12]. In addition, previous studies investigated the dependence of specificity on the type of mismatched nucleotide and position of the mismatch [13,14]. However, as these experimental studies were performed using samples spiked into the transcriptome, *i.e*., mixtures of thousands of transcripts, a certain amount of cross-hybridization is inevitable. Thus, in such analyses, quantification of a small difference in signal intensity between PM and MM probes can be difficult due to the presence of cross-hybridization, and thus evaluation of specificity for single nucleotide mismatches is difficult at low target concentrations.

In the present study, to quantify the specificity for single nucleotide mismatches, we (i) designed a set of artificial random 25-mer sequences, (ii) synthesized oligodeoxyribonucleotides of these random sequences as targets, and (iii) designed a custom microarray with PM probes completely matching the oligodeoxyribonucleotides and MM probes considering all possible single substitutions, *i.e*., all possible one-base substitutions for all possible positions. The use of artificially synthesized oligodeoxyribonucleotides only allows us to quantify the absolute signal intensity without the effect of cross-hybridization and then to evaluate the specificity for single nucleotide mismatches even when the applied target concentration is low. Another advantage of the use of oligodeoxyribonucleotides as targets is that we can analyze the specificity of single nucleotide mismatches without effects of target variation, such as variations in target length due to random fragmentation [15,16]. Furthermore, to evaluate the effects of probe length and position of mismatch on the hybridization behavior, we designed a custom microarray with PM and MM probes of several different lengths from 14- to 25-mer and all possible single mismatches.

Using this custom array, we investigated how the specificity for single nucleotide mismatches depends on the probe length and mismatch position. Our results indicated that, under standard hybridization conditions, the specificity for single nucleotide mismatches becomes maximal at 19~21-mer, which is shorter than the length used on popular high-density oligonucleotide microarrays. With regard to the mismatch position, we confirmed that the specificity with a single nucleotide mismatch decreases at both ends of the probe, as reported previously [13,14,17,18]. In these analyses, as the conditions without the source of cross-hybridization are quite different from those of standard microarray analysis, we performed the experiments with the source of cross-hybridization by adding a mixture of cDNAs generated from *Escherichia coli *total RNA. The same results were obtained, which indicated the possibility of improving measurements of gene expression and genome sequence by microarray analysis by reducing the probe length.

## Results

### Design of the array

To investigate the effects of probe length and position of mismatch for target-specific hybridization comprehensively on a high-density oligonucleotide array, we designed a custom array on which a number of probes were arranged in length, mismatch position, and types of mismatched nucleotide, using Maskless Array Synthesizer platform with the Affymetrix NimbleExpress program [19,20].

**Figure 1:**
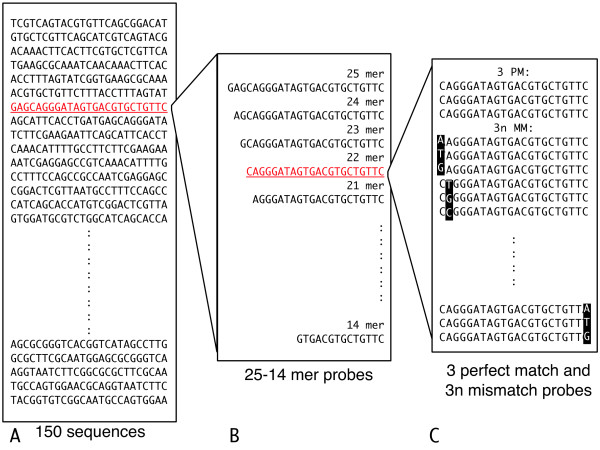
**Scheme for the design of the custom array**. The sequences complementary to the artificially synthesized 25-mer oligodeoxyribonucleotides are shown on the left (A). The sequence highlighted in red is representative for the diversity of probe length as follows. Middle sequences (B) are variant probes the length of which is changed from 25- to 14-mer. In each 25-mer probe, the original sequence is shortened progressively by one nucleotide at the 5' terminal end. The sequence highlighted in red is representative for the diversity of mismatch position and types of mismatched nucleotide as follows. Sequences on the right (C) show three perfect match probes and all possible single base substitutions arranged at each nucleotide.

Figure 1 shows our scheme for the design of the custom array. First, we randomly generated 150 probe sequences 25 nucleotides in length. The range of hybridization free energy of these probes, estimated by the nearest neighbor model [21], was set to meet the conditions of the free energy distribution of probes on the Affymetrix *E. coli *Antisense Genome Array. To avoid cross-hybridization between these probes and targets, we arranged for the maximal sequence overlap among these probes to be less than 6 nucleotides.

For each basic 25-mer probe described above, we generated various probes by changing their length, mismatch position, and type of mismatched nucleotide as follows. First, we initially shortened the probe length by one nucleotide from 25- to 14-mer at the 5' end, to investigate the optimum probe length (Fig. 1 middle sequences). Second, we arranged 3 + 3n probes for a probe in each n-mer length. The first three probes were the same and complementary to the target sequences, which corresponded to perfect match (PM) probes. In the remaining 3n probes, we provided all possible substitutions – *i.e*., each mismatch position and each type of mismatched nucleotide (Fig. 1 right sequences) (note that the design of mismatch (MM) probes on this array was not equivalent to that of Affymetrix catalog GeneChip. We use this term for all these probes with nucleotide substitutions). Collectively, there were 738 probes of various sequences (36 PMs and 702 MMs) for each basic 25-mer probe sequence. Sequences of all probes are given in additional file 1.

### Effects of probe length on signal intensity

To characterize the absolute signal intensities of PM and MM probes, artificially synthesized 25-mer oligodeoxyribonucleotides complementary to the PM probes were applied to the custom array. The target oligodeoxyribonucleotides were applied in tenfold dilutions such that the target oligodeoxyribonucleotides would yield final concentrations of 1.4 nM to 1.4 fM. The use of synthesized oligodeoxyribonucleotides only as hybridization target enabled us to analyze the precise behavior of hybridization without the effect of cross-hybridization. In fact, the signal intensities of PM probes to which no complementary target oligodeoxyribonucleotides were applied were much lower and negligible compared to those of PM probes to which the targets were added in all ranges of oligodeoxyribonucleotides concentration used (data not shown).

**Figure 2:**
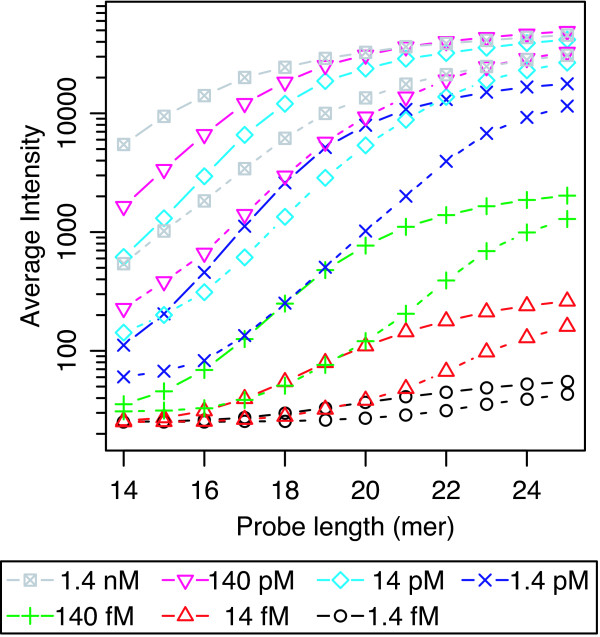
**Signal intensities of probes**. Signal intensities of random probes as a function of probe length in the 7 levels of target oligodeoxyribonucleotide concentration range of 1.4 fM to 1.4 nM. The signal intensities of PM probes were averaged over three PM probes and those of MM probes are represented by the average of the mismatch probes the center bases of which were substituted to all other nucleotides. Solid and dashed lines correspond to signal intensities of PM and MM probes, respectively. Standard deviations are given in additional file 2.

Figure 2 shows the average signal intensities of 150 PM probes and those of corresponding MM probes as a function of the probe length. The signal intensities of MM probes were calculated as the averages of the intensities of mismatch probes the center nucleotides of which were substituted to the other three nucleotides. As addition of error bars clutters the figure, standard deviations are given in additional file 2. In addition, supplemental figures are separated into 7 figures with respect to each target concentration. Hereafter, standard deviations are provided only in additional files (see additional files 2, 3, 4, 5, 6). The changes in PM and MM probe intensities with respect to the probe length exhibited a typical sigmoidal shape, as expected due to the decrease in hybridization free energy with increasing probe length. The decrease in MM probe intensities compared to those of PM probes represents the difference in hybridization free energy to the corresponding target oligodeoxyribonucleotides, which is caused by the mismatched nucleotide at the center of the MM probes. An important point to note is that the signal intensities of longer PM probes (23- to 25-mer) were saturated in both higher and lower target concentrations. In the range of higher target concentrations (14 pM-1.4 nM), saturation occurs due to the finite availability of probe molecules. That is, all probe molecules on a spot were hybridized to corresponding labeled target molecules in this concentration range. On the other hand, the signal intensities of longer PM probes were also saturated in the range of relatively low concentrations (1.4 fM-1.4 pM). This saturation at lower concentrations cannot be explained under the assumption that the number of target oligodeoxyribonucleotide molecules in the hybridization solution is much larger than that of corresponding probe molecules, which is a basic assumption made in several previous studies on microarray analysis [22,23]. When target oligodeoxyribonucleotide molecules remain in the hybridization solution in sufficient numbers, the signal intensity should increase with decreasing hybridization free energy, the decrease of which is approximately proportional to the probe length. In this range of target concentration, the observation that the saturation level of PM probe intensities changes in accordance with the applied target concentrations (*i.e*., tenfold dilution series) strongly suggests that this saturation is due to the finite availability of target oligodeoxyribonucleotide molecules in the hybridization solution. Note that in the range of longer probes (23- to 25-mer), as the signal intensities of PM probes were saturated in all concentration ranges, the differences in intensity between PM and MM probes became smaller than those of shorter probes (*e.g*., 19- to 21-mer).

### Effects of probe length on specificity

The MM probe was designed to quantify the signal intensities of cross-hybridization embedded within the PM signal. In the standard protocol in the Affymetrix GeneChip system, to measure the amounts of complementary DNA/RNA molecules hybridized to PM probes, the MM probe signal was subtracted from that of the PM probe to compensate for cross-hybridization. The background concept of the MM probe is that the amount of cross-hybridization to the PM and MM probe pair is nearly identical, although the specific hybridization between intact target and MM probe is expected to be less due to the mismatched base pairing. Therefore, in this procedure, a pair of PM and MM probes with high specificity for single nucleotide mismatch is desirable, as a small difference in intensity between PM and MM probes can be overcome easily by experimental error and the difference in amount of cross-hybridization between the PM and MM probe.

**Figure 3:**
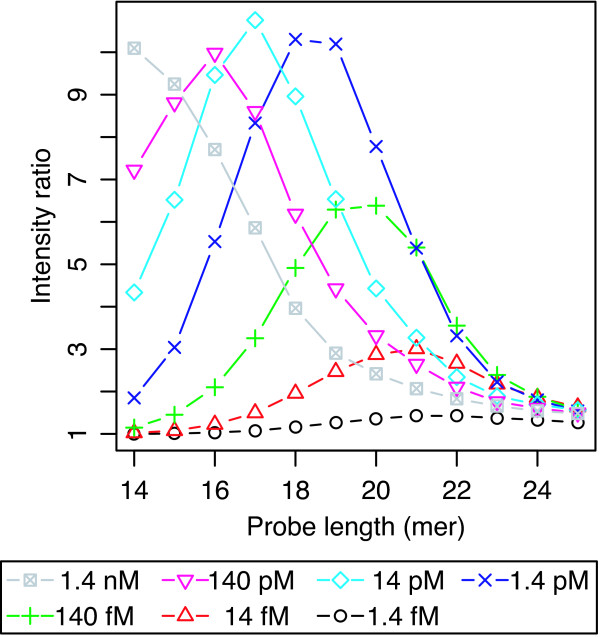
**Ratios of signal intensity of PM to those of cognate MM probes**. The PM/MM ratios as a function of probe length in the 7 levels of target oligodeoxyribonucleotide concentration range of 1.4 fM to 1.4 nM. The averaged signal intensities were derived from three replicate GeneChip analyses and over all 150 signal intensities of probes of the same length. Standard deviations are given in additional file 3.

Figure 3 shows the ratios of signal intensity of PM to those of corresponding MM probes as a function of probe length. The PM/MM signal intensity ratio is an index of specificity [12,24,25] and is especially important when focusing on differences in the sequences of targets, *e.g*., in the case of re-sequencing experiments for analysis of genetic variation [26]. As shown in the figure, the PM/MM signal intensity ratios of the relatively longer probes became smaller due to saturation of PM signal intensities in the longer probes, as described above. This result indicates that, in analysis based on the intensity differences between PM and MM probes, longer probes (23- to 25-mer) suffer from low specificity for single nucleotide mismatch due to the saturation of PM signals.

### Effects of mismatch position and type of mismatched nucleotides on specificity

**Figure 4:**
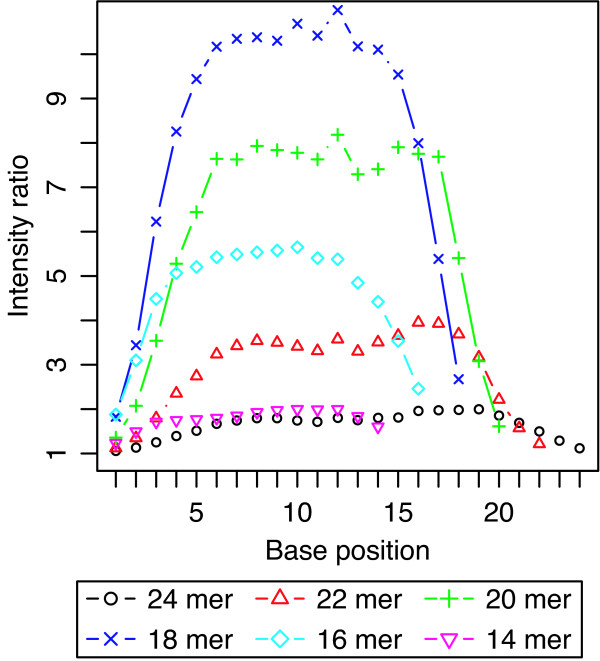
**The effects of the mismatch position on the PM/MM ratio**. The effects of the mismatch position of representative 6 probe lengths on the ratios of signal intensity of PM to those of cognate MM random probes in the target concentration of 1.4 pM. The x-axis denotes the mismatch position as position 1 indicates the first base at the 3' terminal end, which was tethered to the surface *via *the linker moiety. Standard deviations are given in additional file 4.

It is known that the position of the mismatched nucleotide affects specificity [13,14,17,18]. As mentioned in the above section "Design of the array," we provided all possible substitutions, *i.e*., each mismatch position and each type of mismatched nucleotide in every probe length, to investigate mismatch conditions comprehensively. Figure 4 shows the effects of mismatch position on the specificity for a single nucleotide mismatch, as determined from the PM/MM ratio, obtained without DNA/RNA background. Several curves indicate the PM/MM ratios for different probe lengths. As the qualitative behavior was common for all target concentrations investigated, we show typical results obtained at a target concentration of 1.4 pM. The results were generally consistent with previous estimations [13,14,17,18] that the specificity on single nucleotide mismatch at both ends of the probes decreases because binding will be more unstable. This implies that the binding efficiency is mostly determined through local interactions of bases, and the instability at the ends of the probes affecting only a few base pairs. It is worth noting that the PM/MM ratios of relatively short (14- to 20-mer) probes show a typical shape with a flat peak. However, those of relatively long (22- and 24-mer) probes were slightly greater at positions slightly out-of-center, particularly on the 5' side, than at the center. This result suggests that a mismatch position slightly out-of-center is better for specificity than that at the center. Recently, it was reported that there is a 5' bias for hybridization effects based on the analysis of the publicly available dataset [27]. Our results also support this 5' end bias. Of particular interest, our results indicated that the effect of mismatched position on the behavior of duplex formation changes according to probe length. This result would provide a more quantitative and consolidate parameters for models based on the position-dependent nearest neighbor method [17].

Next, we investigated the effects of the type of mismatched nucleotide on specificity, as shown in Table 1. The results indicated that the PM/MM ratio does not change markedly. However, A-G and G-A mismatches decrease the PM/MM ratio slightly. This result is generally consistent with those of previous reports [14,28]. The average intensity ratio of PM probes to each MM probe type correlates roughly with the cost of mismatch estimated using the nearest neighbor model [29,30]. Detailed analysis of these behaviors will enable us to improve the nearest neighbor model on an oligonucleotide array.

**Table 1:** Effects of type of nucleotide mismatch on the PM/MM ratio

	**Probe**			
	
**Target**	**A**	**T**	**G**	**C**
A	3.54 (3.17)	-	2.38 (2.27)	3.61 (3.15)
T	-	2.91 (2.75)	3.09 (2.90)	3.79 (3.25)
G	2.25 (2.18)	2.64 (2.46)	3.33 (2.90)	-
C	3.55 (3.07)	3.65 (3.14)	-	3.93 (3.10)

Effects of type of nucleotide mismatch on the ratios of signal intensity of PM to those of cognate MM probes in the target concentration of 1.4 pM. The signal intensities of PM probes were averaged over probes of all lengths. Those of MM probes with mismatches in all positions were grouped by type of nucleotide mismatch and averaged for the group. The signal intensity ratios are expressed as geometrical averages for each type of nucleotide mismatch. Values in parentheses represent standard deviations. To distinguish types of nucleotide mismatch, the types of nucleotide of the target and probe are indicated in rows and columns, respectively.

### Effects of background cDNA

**Figure 5:**
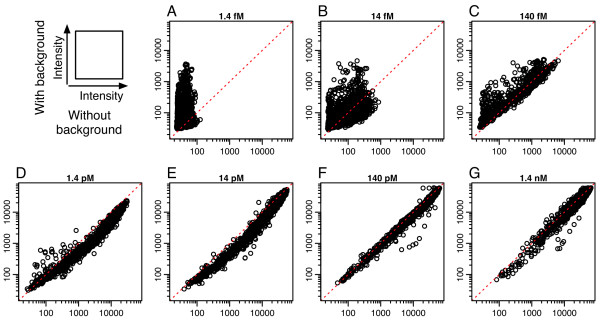
**The comparison of signal intensities with and without cDNA background**. Log-log scatter plots comparing the signal intensities of PM random probes with and without background cDNA generated from *E. coli *total RNA in the 7 levels of target oligodeoxyribonucleotide concentration range of 1.4 fM to 1.4 nM. The x- and y-axes denote the signal intensities of PM random probes of all lengths without and with cDNA background, respectively.

Although we hybridized only target oligodeoxyribonucleotides without any additional source of cross-hybridization, these were quite different from the standard conditions for analyses of genome-wide gene expression and genetic variation. To validate our findings under standard hybridization conditions, we hybridized the target oligodeoxyribonucleotides in the presence of a complex cDNA background generated from *E. coli *total RNA as a source of cross-hybridization. Figure 5 shows a comparison of signal intensities with and without background cDNA. In the low target oligodeoxyribonucleotide concentration range of 1.4 fM to 140 fM (Figure 5A–C), most of the signal intensities with cDNA background were larger than those without cDNA background. This increase was derived from cross-hybridization because the signal intensities from hybridization of the specific target were very small in the low target concentration (Figure 2). However, in the mid-range target concentration of 1.4 pM and 14 pM (Figure 5D,E), the signal intensities with cDNA background were smaller than those without cDNA background. One possible cause of this signal reduction may be target-target interactions in the hybridization solution. Halperin *et al*. [31] and Binder [32] reported that competitive hybridization of target to other target molecules in the bulk solution may decrease the concentration of free target molecules. These results suggest that there are two types of cross-hybridization, *i.e*., "cross-hybridization to probe" and "cross-hybridization to target." The former increases the signal intensity of each probe, while the latter reduces the signal intensity.

**Figure 6:**
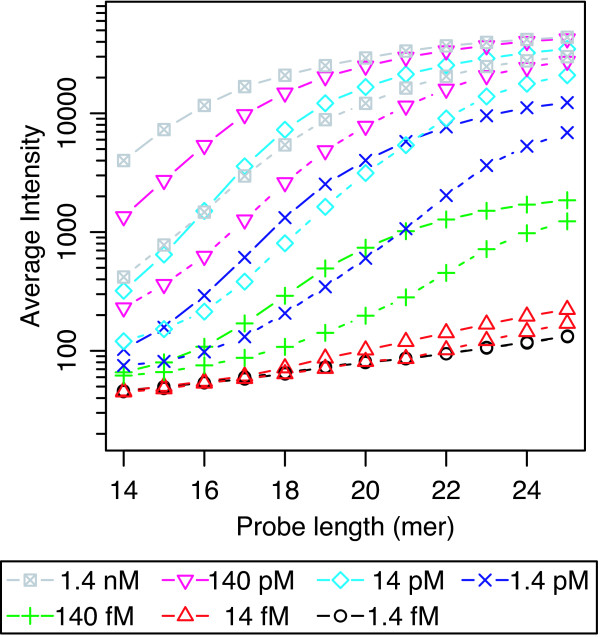
**Signal intensities of probes with background cDNA**. Signal intensities of random probes with background cDNA generated from *E. coli *total RNA as a function of probe length in the 7 levels of target oligodeoxyribonucleotide concentration range of 1.4 fM to 1.4 nM. The signal intensities were determined using GCOS 1.0 software (Affymetrix). The signal intensities of one of the three PM probes and those of MM probes are represented by the averages of the mismatch probes the center bases of which were substituted with all other nucleotides. The signal intensities are plotted on a log(10)-scale. Standard deviations are given in additional file 5.

**Figure 7:**
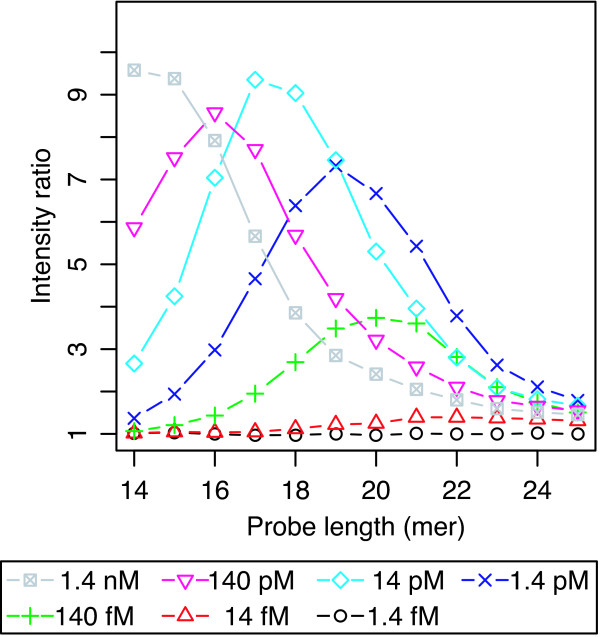
**Ratios of signal intensity of PM to those of cognate MM probes with background cDNA**. Ratios of signal intensity of PM to those of cognate MM random probes with background cDNA as a function of probe length in the 7 levels of target oligodeoxyribonucleotide concentration range of 1.4 fM to 1.4 nM. The signal intensities were determined using GCOS 1.0 software (Affymetrix). The averaged signal intensities were derived from signal intensities of all 150 probes of the same length. Standard deviations are given in additional file 6.

**Figure 8:**
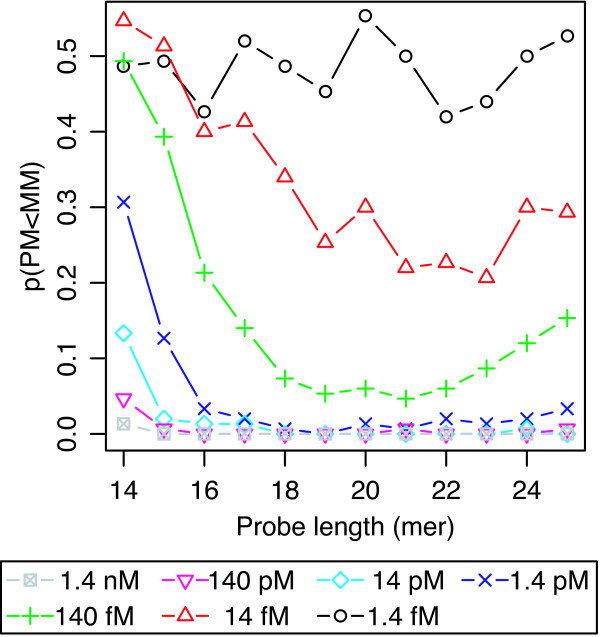
**The reversal probability with background cDNA**. The probability of reversal of signal intensities between PM and MM probes, *i.e*., MM probes possess greater fluorescence intensity than their cognate PM probes (p(PM < MM)) with background cDNA. The signal intensities of one of the three identical PM probes were chosen at random, and MM probes the center bases of which were substituted with the complementary nucleotides were used in this analysis.

The addition of cDNA background did not change the specificity for the single nucleotide mismatch presented in the previous sections. Figure 6 shows the averaged signal intensity of 150 PM probes and those of corresponding MM probes as a function of probe length, obtained with cDNA background. Again, the results showed that the signal intensities of longer probes (*i.e*., 23- to 25-mer) were saturated in both high and low concentrations of target oligodeoxyribonucleotide. The specificity for single nucleotide mismatches measured by the PM/MM ratio decreased with such longer probes (Figure 7), as in the case without cDNA background (Figure 3). The observation that such longer probe pairs have low specificity for single nucleotide mismatch suggests that measurements using a pair of longer PM and MM probes with low specificity may suffer from experimental errors arising from the cDNA background. To check this possibility, we investigated the probability that the MM signal has larger intensity than that of the corresponding PM probe, *i.e*., p(I_PM _< I_MM_), as a function of probe length. It is well known that the reversal of PM and MM probe intensities disturbs the accurate estimation of DNA/RNA amount if not properly analyzed, *e.g*. in MAS5.0 [5,6]. As shown in Figure 8, at lower target concentrations (*i.e*., 14 fM and 140 fM), the probability p(I_PM _< I_MM_) becomes minimum in the mid-range of the probe length, while the probability increases with increasing probe length. This result is consistent with the decrease of specificity for single nucleotide mismatch with increasing probe length, as shown in Figures 3 and 7.

## Discussion

In this study, the results showed that the saturation of intensities occurs even in the low target concentration range of 14 fM and 140 fM, probably due to the finite availability of target molecules in the hybridization solution. It was described in the Expression Analysis Technical Manual (Affymetrix, 2004) that the number of probe molecule contained in each probe cell is on the order of 10^6^, which is approximately comparable that of the target molecules at about 10 fM. In this study, the finite availability of target molecules was observed even at 1.4 pM. These observations suggest that the effective amounts of target molecules are decreased due to nonspecific hybridization to other probes, non-biospecific adsorption on the array surface, competitive hybridization between the probes that share the same target, etc. Thus, the effect of finite availability cannot be neglected when we measure the target of low concentration quantitatively. The results indicated that, in this microarray system, as long as the analysis is based on the intensity change by single nucleotide mismatch, the longer probes (>23-mer) are not suitable for accurate measurement of cDNA/cRNA amount due to their low specificity. On the other hand, when we use shorter probes for genome-wide analysis, it should be take into account that probes that are too short may match too many subsequences in the genome. To check the uniqueness of an oligodeoxyribonucleotide sequence within the whole genome sequence, we sampled random subsequences from the *E. coli *genome, and searched for their matching alignments throughout the genome. When we changed the length of sample subsequences from 14- to 25-mer, both the number of matching copies and the probability that a probe sequence is not unique increased markedly below 15-mer, while more than 97% of sequences were unique at longer than 18-mer (data not shown). Thus, the optimal probe length for the specificity for single nucleotide mismatches is 19–21-mer, which was also supported by the probability of reversal of PM and MM probe intensity p(I_PM _< I_MM_) as shown in Figure 8. The observation that optimization of probe length had a marked impact on the specificity for single nucleotide mismatches is important to improve probe design for accurate analysis of gene expression and genetic variation on microarrays. Of course, the experimental conditions in the present study were different in several respects from those of typical microarray experiments. For example, in standard genome-wide analysis of gene expression, cRNA targets into which biotinylated ribonucleotides are incorporated are randomly fragmented to 50–200 bases in length. That is, the standard target samples have dangling ends varying in both length and sequence not hybridized to the probes. Recently, it has been reported that the dangling ends reduce the specificity of probe-target hybridization [33]. This report suggests that the fragmentation pattern has an influence on the accuracy of typical GeneChip analysis. Therefore, further studies are necessary to optimize probe length for accurate measurement of DNA/RNA amounts under the standard conditions used in genome-wide analyses of gene expression and genetic variation.

Cross-hybridization is problematic for GeneChip analyses because it adds background intensity, which is not related to the true amounts of target DNA/RNA. Although MM probes are provided on GeneChips to evaluate the amount of cross-hybridization, GeneChip analyses have shown that a number of MM probes possess greater fluorescence intensity than their cognate PM probes [5,6]. A previous study indicated that the reversal of PM and MM probe intensities was due to cross-hybridization [24]. As shown in Figure 8, the probability p(I_PM _< I_MM_) increases significantly as the target oligodeoxyribonucleotide concentration decreases. Especially at the lowest target concentration of 1.4 fM, the probability reached 0.5. These results also suggested that the increase in probability was caused by cross-hybridization, because the relative amount of cross-hybridization increases with decreasing target concentration. These findings clearly indicated that the use of MM probes for assessment of cross-hybridization is unreliable. Therefore, data analyses have been carried out without using the signal intensities of MM probes in Robust Microarray Analysis (RMA), which is one of the most commonly used algorithms for GeneChip systems [8,9]. On the other hand, our findings suggested that a well-designed probe would enable us to make efficient use of MM probes in GeneChip data analysis. Thus, it would be possible to achieve further improvement of the algorithms for GeneChip systems.

As shown in Figure 4, we found that the PM/MM ratios of relatively long (22- and 24-mer) probes were slightly greater at positions slightly out-of-center, particularly on the 5' side, than at the center. Although the 5' bias was thought to be due to the array surface, the observation that slightly out-of-center mismatches provide better specificity than those at the center is puzzling. Although several factors may influence this observation, such as steric hindrance and synthetic errors of probes, one possible cause of this phenomenon may be a cooperative relationship of instabilities between the ends of probes and the mismatched base pair. That is, each unstable end and mismatched base pair may destabilize hybridization of three or four neighboring base pairs, preventing hybridization of whole base pairs between the end and mismatched position. Although this speculation is also supported by the observation that the PM/MM ratios of the mismatched positions 6–8 from the end showed the best specificity with most probe lengths, further studies are necessary to understand the effects of the mismatched position on hybridization behavior on microarrays.

In the present study, we used the standard conditions for hybridization and washing processes generally used in the GeneChip system and did not address the effects of hybridization temperature and stringency. It has been shown that hybridization conditions affect the specificity for single nucleotide mismatch, such as hybridization temperature [34], time [35,36], washing stringency [37,38], and dimethylsulfoxide, formamide, etc., included in hybridization solution [39]. Certainly, a change in hybridization conditions can result in a change in optimal probe length on a high-density oligonucleotide array. An important point of our study was that longer probes that show strong hybridization can cause saturation of signal intensities in the range of both high and low target concentrations, which results in low specificity for single nucleotide mismatch. This factor of saturation should be considered in the optimization process of microarray analysis by changing hybridization conditions.

## Conclusion

We designed a custom array on which probes were arranged according to length, mismatch position, and type of mismatched nucleotide to investigate the specificity for single nucleotide mismatch, which is important both for gene expression analysis and mutation analysis in microarray experiments. We applied only oligodeoxyribonucleotides as targets to characterize the probe-target specific hybridization without the effects of cross-hybridization. Using this custom array, we investigated empirically how the specificity for single nucleotide mismatch depends on the probe length and mismatch position by measuring both PM and MM intensities. The results showed that the specificity for single nucleotide mismatch is generally lower in the case of relatively longer probes (23- to 25-mer) than shorter probes. This is due to saturation of signal intensities in the case of such longer probes in all ranges of applied target oligodeoxyribonucleotide concentration.

The dependency of specificity on the position of the mismatch in the probe will allow us to improve the existing position-dependent nearest neighbor model for more precise estimation of binding affinity. Carlon and Heim proposed a thermodynamic theoretical model of oligonucleotide hybridization and explained the behavior of MM probes by taking into account the mismatch penalty on binding free energy [40]. We are now extending the existing nearest neighbor model on microarray to explain these behaviors more correctly. In addition, we also observed that target-target interaction would reduce the concentration of free target in solution. Although it is well known that cross-hybridization to probe increases the signal intensity, it should be considered that cross-hybridization to target can reduce the signal intensity. The adapted hybridization model on microarray and detailed analysis based on this model will be published shortly. Further investigation along this line will facilitate a fundamental understanding of the behavior of microarray probes and provide a promising method to improve the precision of measurement of gene expression levels.

## Methods

### Preparation of biotin-labeled target oligodeoxyribonucleotides

One hundred fifty 25-mer oligodeoxyribonucleotides were synthesized with sequences complementary to the probes of random sequences set as targets. The sequences of 150 oligodeoxyribonucleotides are given in additional file 7. Titration experiments were performed in three technical replicates using three different biotin-labeled target oligodeoxyribonucleotides separately prepared for each sample. This helped minimize technical noise associated with oligodeoxyribonucleotides labeling efficiency. Although the methods for target preparation described in the Expression Analysis Technical Manual (Affymetrix, 2004) were followed, each method for target preparation differed slightly from the others. Briefly, for data set 1, aliquots of 100 pmol of each synthetic oligodeoxyribonucleotides target were labeled independently at the 3' end with 0.3 mM GeneChip DNA Labeling Reagent (Affymetrix) using 60 U of Terminal Deoxynucleotidyl Transferase, Recombinant (TdT; Promega, Madison, WI) at 37°C for 1 h. After TdT had been stopped by addition of EDTA to a final concentration of 9.6 mM, the 150 labeled oligodeoxyribonucleotides were mixed. As the results derived from data set 1 showed that the signal intensities corresponding to four target oligodeoxyribonucleotides, 012, 064, 072, and 091, were anomalous, we re-synthesized these four oligodeoxyribonucleotides. For data set 2, we replaced the anomalous target oligodeoxyribonucleotides with new oligodeoxyribonucleotides and labeled 150 target oligodeoxyribonucleotides as described above. For data set 3, 150 synthesized oligodeoxyribonucleotide targets were mixed before terminal labeling. The total of 100 pmol of 150 mixed targets (0.67 pmol each) was labeled as described above. Use of the Terminal deoxynucleotidyl Transferase (TdT) end labeling method canceled out fluctuations in labeling efficiency depending on the target sequences caused by *in vitro *transcription using biotinylated UTP and/or CTP, because the activity of TdT does not depend on the sequence of the target [41,42]. Therefore, the efficiency of labeling was the same among the target oligodeoxyribonucleotides.

### Preparation of biotin-labeled background of prokaryotic transcripts

For all experiments that included background cDNA as a source of cross-hybridization, aliquots of 10 μg of *E. coli *total RNA were used. Briefly, *E. coli *K-12 strain W3110 was grown overnight with shaking at 37°C in 5 ml of liquid Luria-Bertani medium. To maintain logarithmic growth, the overnight cultures were diluted to an optical density at 600 nm of 0.05 in 5 ml of fresh liquid Luria-Bertani medium. Then, cultures were grown with shaking at 37°C to an optical density at 600 nm of 0.8. Cells were harvested by centrifugation and stored at -80°C prior to RNA extraction. Total RNA was isolated and purified from cells using an RNeasy mini kit with on-column DNA digestion (Qiagen, Hilden, Germany) in accordance with the manufacturer's instructions. For preparation of cDNA background samples, standard methods for cDNA synthesis, fragmentation, and end-terminus biotin labeling were carried out in accordance with the Affymetrix protocols. Titration experiments with cDNA background were performed in duplicate using different biotin-labeled target oligodeoxyribonucleotides and cDNA background prepared separately for each sample.

### Hybridization, washing, staining, and scanning

Hybridization, washing, staining, and scanning were carried out according to the Expression Analysis Technical Manual (Affymetrix). Briefly, the 150 labeled target oligodeoxyribonucleotides were diluted in hybridization cocktail containing 1× manufacturer's recommended buffer (100 mM MES, 1 M NaCl, 20 mM EDTA, and 0.01 Tween-20), 50 pM B2 Control Oligo, 0.1 mg/mL herring sperm DNA, and 0.5 mg/mL BSA, such that each labeled target oligodeoxyribonucleotide would yield final concentrations ranging from 1.4 fM to 1.4 nM in tenfold dilutions. In the experiment that included cDNA background, 3 μg of labeled cDNA was added to the hybridization cocktail. The labeled and diluted target oligodeoxyribonucleotide samples with or without background cDNA were hybridized to our custom microarrays at 45°C for 16 h in a Hybridization Oven 640 (Affymetrix) set at 60 rpm under standard conditions. After hybridization, a Fluidics Station 450 (Affymetrix) was used for the washing and staining procedures with ProkGE_WS2_450 fluidics script (Affymetrix) under standard conditions. Following washing and staining, the arrays were scanned using a GeneChip Scanner 3000 (Affymetrix). Absolute signal intensities of every probe in every sample were generated using GCOS 1.0 software (Affymetrix). The raw signal intensities of all probes for each experiment are given in additional file 8.

### Data analysis

The extracted GeneChip data were analyzed using R software [43]. The signal intensities were replicated very well among the three replicates (correlations were 0.98~0.99). The signal levels were computed by taking the arithmetic mean of the three replications on a log scale.

## Authors' contributions

SS performed microarray experiments and drafted the manuscript. NO performed data analysis and drafted the manuscript. CF designed the custom microarray and rewrote the manuscript. AK assisted microarray experiments. TY conceived and supervised the study. All authors reviewed and approved the final manuscript.

## Supplementary Material

Additional file 1 Sequences of all probes. All probe sequences are shown in the 5' to 3' direction. The ID column shows probe IDs, which are identical to those in Table S3. Click here for file

Additional file 2 Signal intensities of probes with error bars. Signal intensities of probes as a function of probe length in the 7 levels of target oligodeoxyribonucleotide concentration range of 1.4 fM to 1.4 nM. Solid lines represent the average intensities of 450 PM probes, *i.e*., 3 copies for each of the 150 oligodeoxyribonucleotides and dashed lines represent that of 450 MM probes, *i.e*., 3 mismatch types for each of the 150 oligodeoxyribonucleotides. Error bars show the standard deviations. Click here for file

Additional file 3 Title of data: Ratios of signal intensity of PM to those of cognate MM probes with error bars. The log intensity ratios (*i.e*., log_10 _(PM/MM)) as a function of probe length in the 7 levels of target oligodeoxyribonucleotide concentration range of 1.4 fM to 1.4 nM. The log intensity ratios are averaged for all 450 probe pairs. Error bars show the standard deviations. Click here for file

Additional file 4 The effects of the mismatch position on the PM/MM ratio with error bars. The effects of the mismatch position of representative 6 probe lengths on the log intensity ratios (*i.e*., log_10 _(PM/MM)) in the target concentration of 1.4 pM. The x-axis denotes the mismatch position as position 1 indicates the first base at the 3' terminal end, which was tethered to the surface *via *the linker moiety. Error bars show the standard deviations. Click here for file

Additional file 5 Signal intensities of probes with background cDNA with error bars. Signal intensities of random probes with background cDNA generated from *E. coli *total RNA as a function of probe length in the 7 levels of target oligodeoxyribonucleotide concentration range of 1.4 fM to 1.4 nM. Solid lines represent the average intensities of 450 PM probes, *i.e*., 3 copies for each of the 150 oligodeoxyribonucleotides and dashed lines represent that of 450 MM probes, *i.e*., 3 mismatch types for each of the 150 oligodeoxyribonucleotides. Error bars show the standard deviations. Click here for file

Additional file 6 Ratios of signal intensity of PM to those of cognate MM probes with background cDNA with error bars. The log intensity ratios (*i.e*., log_10 _(PM/MM)) with background cDNA as a function of probe length in the 7 levels of target oligodeoxyribonucleotide concentration range of 1.4 fM to 1.4 nM. The log intensity ratios are averaged for all 450 probe pairs. Error bars show the standard deviations. Click here for file

Additional file 7 The sequences of 150 synthesized 25-mer oligodeoxyribonucleotide as targets. All sequences of oligodeoxyribonucleotides are shown in 5' to 3' direction. Click here for file

Additional file 8 The raw signal intensities of all probes for each experiment. The ID column shows probe IDs, which are identical to those in Table S1. Click here for file
